# Identification of Blood Transport Proteins to Carry Temoporfin: A Domino Approach from Virtual Screening to Synthesis and In Vitro PDT Testing

**DOI:** 10.3390/pharmaceutics15030919

**Published:** 2023-03-11

**Authors:** Alessia Marconi, Giulia Giugliano, Matteo Di Giosia, Tainah Dorina Marforio, Michele Trivini, Eleonora Turrini, Carmela Fimognari, Francesco Zerbetto, Edoardo Jun Mattioli, Matteo Calvaresi

**Affiliations:** 1 Dipartimento di Chimica “Giacomo Ciamician”, Alma Mater Studiorum—Università di Bologna, Via Francesco Selmi 2, 40126 Bologna, Italy; alessia.marconi5@unibo.it (A.M.); giulia.giugliano2@unibo.it (G.G.); matteo.digiosia2@unibo.it (M.D.G.); tainah.marforio2@unibo.it (T.D.M.); michele.trivini@studio.unibo.it (M.T.); francesco.zerbetto@unibo.it (F.Z.); 2 Dipartimento di Scienze per la Qualità della Vita, Alma Mater Studiorum—Università di Bologna, Corso d’Augusto 237, 47921 Rimini, Italy; eleonora.turrini@unibo.it (E.T.); carmela.fimognari@unibo.it (C.F.)

**Keywords:** docking, virtual screening, MD simulations, MM-GBSA, temoporfin (mTHPC), apomyoglobin, apohemoglobin, blood transport proteins, hemopexin, photodynamic therapy (PDT)

## Abstract

Temoporfin (mTHPC) is one of the most promising photosensitizers used in photodynamic therapy (PDT). Despite its clinical use, the lipophilic character of mTHPC still hampers the full exploitation of its potential. Low solubility in water, high tendency to aggregate, and low biocompatibility are the main limitations because they cause poor stability in physiological environments, dark toxicity, and ultimately reduce the generation of reactive oxygen species (ROS). Applying a reverse docking approach, here, we identified a number of blood transport proteins able to bind and disperse monomolecularly mTHPC, namely apohemoglobin, apomyoglobin, hemopexin, and afamin. We validated the computational results synthesizing the mTHPC-apomyoglobin complex (mTHPC@apoMb) and demonstrated that the protein monodisperses mTHPC in a physiological environment. The mTHPC@apoMb complex preserves the imaging properties of the molecule and improves its ability to produce ROS via both type I and type II mechanisms. The effectiveness of photodynamic treatment using the mTHPC@apoMb complex was then demonstrated in vitro. Blood transport proteins can be used as molecular “Trojan horses” in cancer cells by conferring mTHPC (i) water solubility, (ii) monodispersity, and (iii) biocompatibility, ultimately bypassing the current limitations of mTHPC.

## 1. Introduction

Photodynamic therapy (PDT) is a minimally invasive therapeutic modality, clinically approved for treating several types of cancer [1,2,3,4]. PDT is based on the use of a drug, called photosensitizer (PS) [1,2,3,4,5], that is activated by light absorption to react with the oxygen normally present in cells/tissues, generating oxidative stress that causes (i) direct cytotoxicity on cancer cells; (ii) damages to the tumor vascular system; (iii) and the stimulation of an immune response [1,2,3,4].

Temoporfin (mTHPC, Foscan^®^) [6,7,8] is among the most promising photosensitizers used in PDT [9]. In Europe, since 2001, mTHPC has been approved for the photodynamic treatment of head and neck squamous cell carcinoma (HNSCC) [6]. Despite its excellent photophysical properties, the poor solubility of mTHPC still represents a challenge in clinical settings. In physiological environments, mTHPC forms aggregates, resulting in the quenching of its excited states and, consequently, in reduced production of ROS and in very poor stability [6,7,8]. The approved formulation of Foscan^®^ is a solution of mTHPC in a mixture of ethanol and propylene glycol, which, however, can cause severe side effects post-injection [10].

Due to the hydrophobic character of mTHPC, numerous studies were aimed to create novel delivery systems based on nanosystems such as polymers, liposomes, and nanoparticles [7,8,11] with the intent to increase solubility and stability in a physiological environment (i.e., third-generation photosensitizers) [12].

Among the different biological and technological challenges posed by nanomaterials in their clinical translation [13,14,15], a key point remains the biocompatibility of the nanocarrier. To address this issue, peptide/protein-based theranostics nanoplatforms were recently proposed for transporting hydrophobic drugs/photosensitizers [16,17,18,19,20,21]. They exhibited high loading and conferred water solubility and biocompatibility to the PSs.

Proteins meet the strict requirements for biomedical usage without further sophisticated design and synthesis because of their biocompatibility, structural diversity, abundance from renewable resources, and considerable uptake by cells [16,17,18,19,20,21].

However, when proteins are used in the form of nanoparticles, some major weaknesses remain: (i) random conjugation of PS, (ii) polydispersity of the nanoparticles, (iii) structural alterations of the protein folding caused by the chemical procedures employed to create the nanoparticles, (iv) cargo leakage in the serum, and (v) degradation of the PDT performances due to PS aggregation inside the nanoparticle and/or dispersion of the PS aggregates.

The ultimate solution is the dispersion of a PS inside a single protein. Apart from conferring to the PS water solubility and biocompatibility, the use of single proteins as supramolecular hosts [22], viz. a “Trojan horse” approach [23], has remarkable advantages: (i) the PS binds in a specific and well-defined binding pocket, (ii) the protein maintains its folding, (iii) the complex is characterized by precise stoichiometry [24].

In addition, the existence of specific recognition processes between the PS and the protein ensures that the PS is maintained in a monomeric state, within a suitable hydrophobic environment, such that the photophysical properties of the PS are maintained, realizing the full potential of the PSs.

Water soluble proteins such as albumins [25,26,27,28,29,30,31,32], lysozyme [22,24,33,34], β-lactoglobulin [35], apomyoglobin [36,37,38,39,40,41,42,43], apohemoglobin [44,45], and others [46,47] have binding pockets that are suitable to bind hydrophobic PS.

Human serum albumin (HSA), which naturally transports hydrophobic compounds in the blood, has been intensively studied as a flexible carrier for drugs and PSs [48,49,50,51].

The quickest route to deliver a drug throughout the body is through the bloodstream.

In the proper conditions, almost any type of compound can be transported by carriers through the circulatory system. In fact, a variety of plasma proteins exist and are already involved in the transport of various types of endogenous compounds. They can potentially be exploited as carriers for PS.

Here, using a reverse docking approach, we identified blood transport proteins able to bind and disperse mTHPC in a physiological environment. We validated the computational results synthesizing a complex between one of the identified proteins (apomyoglobin) and mTHPC, demonstrating its ROS-generating properties and in vitro PDT effect.

## 2. Materials and Methods

### 2.1. Computational Details

#### 2.1.1. Blood Transport Proteins Structural Database

Following the classifications of the blood proteins proposed by Schaller [52], all the crystal structures of the blood transport proteins available in the Protein Data Bank (PDB) [53] were downloaded. When the crystal structure of the protein is not available, such as in the case of hemopexin and α-fetoprotein, the protein structure predicted with AlphaFold [54] was used. Before their use in the calculations, water molecules, ions, and co-crystallized ligands were removed from the protein structures. This dataset was used for ensemble docking calculations.

#### 2.1.2. Docking

Ensemble docking calculations were carried out using the mTHPC ligand and the blood transport proteins structural database. The docking poses were obtained using the PatchDock algorithm [55]. FireDock [56] was used to refine (rearranging the side-chains of amino acids close to the ligand and adjusting the relative orientation of the molecules) and rescore the obtained poses.

#### 2.1.3. MD Simulations

Proteins were described by the Amber ff14SB force field; [57] the atomic charges of mTHPC were calculated with the Merz-Singh-Kollman scheme; whereas for the rest, mTHPC was parametrized using the GAFF force field [58]. The TIP3P water model was used for all simulations, and Na^+^ or Cl^−^ counterions were introduced to neutralize the system. The particle mesh Ewald summation and periodic boundary conditions (PBC) were applied throughout (with a cut-off radius of 10.0). A time step of 2 fs was chosen for all MD runs, and the SHAKE algorithm was used for H-atoms. The systems were minimized and equilibrated, and then 100 ns of MD simulations were carried out.

#### 2.1.4. Molecular Mechanics-Generalized Born Surface Area (MM-GBSA) Analysis

Using CPPTRAJ [59], one frame every 0.1 ns was retrieved from the MD trajectories and used as input for the MM-GBSA analysis. MM-GBSA analysis was carried out with the MMPBSA.py [60] module, determining the binding affinity between mTHPC and the different proteins. Electrostatic and van der Waals (vdW) interactions were calculated considering an infinite cut-off. The polar solvation term was calculated using the Generalized Born (GB) model, whereas the non-polar solvation term was determined using solvent-accessible, surface-area-dependent terms.

### 2.2. Synthesis and Characterization of the mTHPC@apoMb Complex

#### 2.2.1. Materials

Myoglobin (Cat. No. M1882); 2-butanone (Cat. No. W217018); hydrochloric acid (Cat. No. 320331); NaOH (Cat. No. 30620); dimethyl sulfoxide (DMSO) (Cat. No. 472301); deuterium oxide (Cat. No. 151882); 9,10-anthracenediylbis(methylene)dimalonic acid (ABMDMA) (Cat. No. 75068); 10-acetyl-3,7-dihydroxyphenoxazine (Amplex Red) (Cat. No. 90101); type VI-A peroxidase from horseradish lyophilized powder (HRP) (Cat. No. P6782); hydrogen peroxide solution 30% (*w*/*w*) (Cat. No. 31642-M); Amicon Ultra centrifugal filters (MWCO 30 kDa, Millipore UFC503024, Cat. No. Z677892-24EA); sodium chloride (Cat. No. S9888-M); potassium phosphate monobasic (Cat. No. P0662-M); sodium phosphate dibasic (Cat. No. S0876); potassium chloride (Cat. No. P3911M); and MWCO 14 kDa dialysis tubing cellulose membrane (Cat. No. D9652) were purchased from Sigma Aldrich (Merck, Darmstadt, Germany). 3,3′,3″,3‴-(7,8-dihydro-21H,23H-porphine-5,10,15,20-tetrayl)tetrakis-phenol (mTHPC) (Item No. 17333) was purchased from Cayman Chemical (Ann Arbor, MI, USA). All the reagents were used without further purification. Milli-Q water was used for the preparation of all the aqueous solutions.

#### 2.2.2. Synthesis of the Reconstituted Myoglobin-Temoporfin Adduct

A standard procedure [41,61] was used to prepare apomyoglobin. HCl 1 M was added dropwise to 4 mL of water solution of myoglobin (90 μM), and the solution was kept under stirring at 0 °C into an ice bath. At pH 2.5, the interaction between the heme and the protein is strongly reduced due to the protonation of its carboxylic groups. The same volume of 2-butanone was added, and the extraction process was repeated three times with fresh 2-butanone. A solution of pure apo-myoglobin was obtained. Then, a solution of mTHPC in 2-butanone (a 2:1 stoichiometry of mTHPC/apoMb was used) was added to the aqueous phase of apoMb. NaOH 5 M was added to reach a pH of 12.5, maintaining the system under gentle stirring. Deprotonation of the phenolic moieties of mTHPC allowed its migration from the organic to the aqueous phase and its quantitative intercalation with the apoMb. The mTHPC@apoMb adduct was extensively dialyzed against PBS, using an MWCO 14 kDa dialysis tubing cellulose membrane to remove 2-butanone and mTHPC in excess. A 1:1 stoichiometry of mTHPC/apoMb was obtained after purification.

#### 2.2.3. Characterization of mTHPC@apoMb

*UV-Vis Spectroscopy.* The solutions of mTHPC, apoMb, and mTHPC@apoMb were characterized through UV-Vis spectroscopy. The absorption spectra were collected using a Cary60 UV-Vis spectrophotometer (Agilent Technologies, Stockport, UK).

*Fluorescence Spectroscopy.* The fluorescence spectra of mTHPC and mTHPC@apoMb were acquired with an Edinburgh FLS920 equipped with a photomultiplier Hamamatsu R928P.

#### 2.2.4. Detection of Reactive Oxygen Species

*ABMDMA assay.* To detect and estimate the amount of singlet oxygen (^1^O_2_) generated by mTHPC and mTHPC@apoMb upon irradiation, the ABMDMA assay was used. ABMDMA reacts with singlet oxygen and this reaction can be followed by monitoring the decrease of the UV absorption bands of the ABMDMA [62,63,64,65].

Solutions of mTHPC and mTHPC@apoMb were exchanged with deuterated PBS. An amount of 97 µL of each sample (mTHPC and mTHPC@apoMb, 1 μM) and 3 µL of ABMDMA 5 mM in DMSO were loaded into the wells of a 96-multiwell plate. The plate was then irradiated using a cold white LED (Valex 30 W, 6500 K), (irradiance 24 mW cm^−2^, energy fluence = 86 J cm^−2^, measured with the photo-radiometer Delta Ohm LP 471 RAD on the plate surface). The absorbance before and after the irradiation was measured at 380 nm using an EnSpire^®^ Multimode Plate Reader (PerkinElmer, Waltham, MA, USA).

*Amplex Red assay.* The Amplex Red assay was used to quantify the peroxides generated by the irradiated samples. It works by monitoring the enzymatic reaction, catalyzed by HRP, that occurs between the nonfluorescent Amplex Red with peroxides, producing fluorescent resorufin [28,62,65,66,67].

An amount of 90 µL of each sample (mTHPC and mTHPC@apoMb, 1 μM) was loaded into the wells of two 96-multiwell plates. One plate was irradiated under the same conditions used for the ABMDMA assay, whereas the other was kept in the dark. A working solution (WS) containing Amplex Red and HRP dissolved in phosphate buffer 50 mM was freshly prepared. An amount of 10 µL of the WS was then added to each well and both plates were kept in incubation for 30 min in dark conditions at room temperature.

The emission of the resorufin was recorded at 590 nm (λ_ex_ 530 nm) using the EnSpire^®^ Multimode Plate Reader (PerkinElmer). The fluorescence values were converted to the H_2_O_2_ concentration using a calibration curve created with standard solutions of H_2_O_2_. The H_2_O_2_ concentration generated by irradiated samples was obtained by subtracting the contribution of the H_2_O_2_ produced by the corresponding samples kept in the dark.

### 2.3. Cytotoxicity and Phototoxicity of mTHPC@apoMb Complex in CAL27 Cells

#### 2.3.1. Cell Line

The HNSCC cells line, CAL27, derived from squamous carcinoma of the oral tongue, was kindly gifted by the laboratory of Experimental Radiotherapy, Leuven, Belgium. The cells were propagated in culture in Dulbecco’s Modified Eagle Medium (DMEM) high glucose, supplemented with 10% heat-inactivated fetal bovine serum, 1% L-glutamine solution 200 mM, and 1% penicillin/streptomycin solution 100 U mL^−1^ (all supplied by Euroclone, Pero, Italy). The cells were cultured in incubator at 37 °C in a 5% CO_2_ humidified atmosphere. To maintain exponential growth, the cells were trypsinized using the cell dissociation solution, Versene (Merck, Darmstadt, Germany), before reaching 80% confluence.

#### 2.3.2. Cell Viability

CAL27 cells were treated in complete medium with increasing concentrations of mTHPC DMSO stock solution diluted in complete medium (mTHPC/CM) or mTHPC@apoMb (0.01–1.00 μM) for 45 min. When the incubation time was over, the cells were washed twice in PBS 1X and irradiated in PBS 1X with white light LED (24 mW cm^−2^) for 45 min. In order to verify possible dark toxicity, the cells were exposed to mTHPC/CM or mTHPC@apoMb while kept in the dark. After the cells were recovered for 24 h in complete medium, the cell viability was determined using a colorimetric MTT (3-(4,5-dimethylthiazol-2-yl)-2,5-diphenyltetrazolium bromide) assay (Merck) [68,69].

## 3. Results and Discussion

### 3.1. Identification of Blood Proteins as Carriers for mTHPC by Virtual Screening

We applied a reverse docking approach to identify blood transport proteins that are suitable to bind mTPHC. Following the classifications of the blood proteins proposed by Schaller [52], we first built a structural database of blood transport proteins, and then we carried out ensemble docking calculations to determine the affinity of mTHPC with the different proteins. To investigate in detail the binding between mTHPC and the most promising proteins, we carried out 100 ns of molecular dynamics (MD) simulation, followed by MM-GBSA analysis. Table 1 presents the proteins that bind the most to mTHPC, as ranked by the reverse docking program employed.

**Figure 1:**
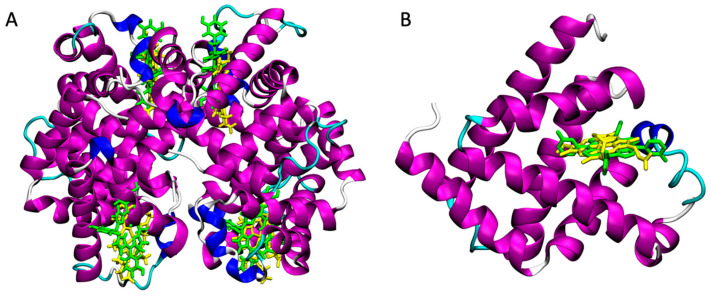
(**A**) Superposition of the crystallographic structure of heme (in yellow, PDB 2m6z) and docked structure of mTHPC (in green) in the heme-binding sites of Hb. (**B**) Superposition of the crystallographic structure of heme (in yellow, PDB 3rgk) and docked structure of mTHPC (in green) in the heme-binding site of Mb.

We recently experimentally demonstrated the possibility to use HSA as a carrier for mTHPC [68]. Docking calculations are commonly used to identify the favourite binding site of a ligand in HSA [70,71]. HSA ranks fifth, which means that, potentially, hemoglobin, myoglobin, hemopexin, and afamin are even more suitable transport proteins for mTHPC. Very interestingly, hemoglobin and myoglobin were already used as a delivery system for PS [36,37,38,39,40,41,42,43,44,45]. In particular, the empty heme-binding pocket of apohemoglobin and hapomyoglobin, obtained via removal of the heme groups, can bind hydrophobic molecules such as PS, maintaining them in a monomeric state and providing water solubility via supramolecular interactions [36,37,38,39,40,41,42,43,44,45]. Docking calculations suggested that the same approach can be used for mTHPC. The docked structures show that mTHPC occupies the heme-binding sites, which are large enough to also host this molecule that is larger than the heme group (Figure 1).

Human hemoglobin (Hb), the main protein found in red blood cells (RBCs), is responsible for the transportation of oxygen (O_2_) and carbon dioxide (CO_2_) in blood. In physiological conditions, Hb is a tetramer (α_2_β_2_, 64 kDa) of noncovalently linked chains, generally two α- and two β-chains. In Hb, the two α subunits and the two β subunits are arranged into two dimeric halves (αβ) [52]. The folding of α and β chains is typical of globin proteins, characterized, respectively, by the presence of seven and eight α-helices. There are 141 amino-acid residues in α-globin and 146 in β-globin. The missing residues correspond to the D helix of β-globin [52]. The helices pack together into a small, tightly-packed globule, which heterodimerizes to create the usual quaternary shape of Hb. Each subunit is characterized by a central hydrophobic pocket, i.e., the heme-binding sites [52].

**Figure 2:**
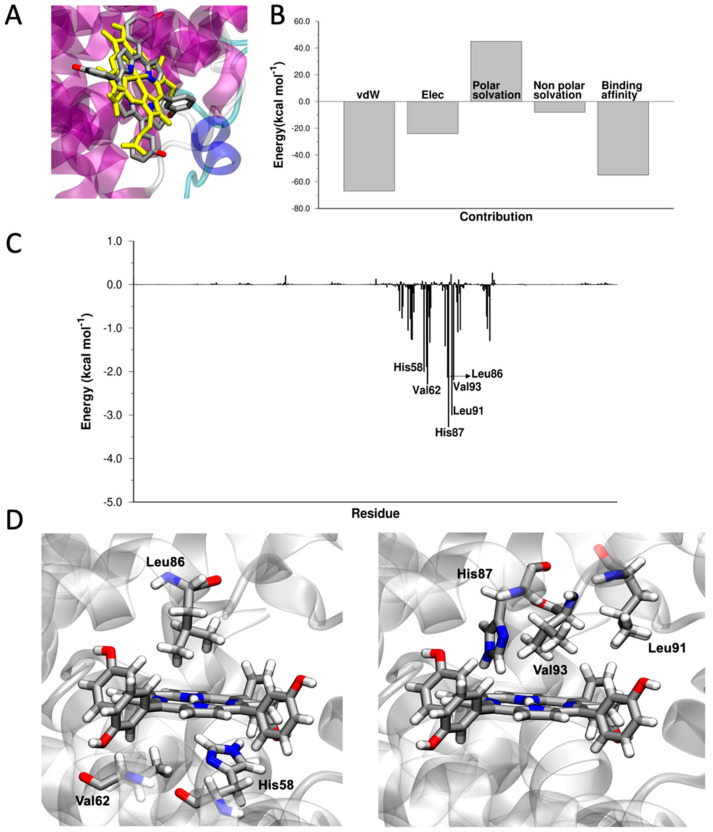
(**A**) Superposition of the crystallographic structure of heme (in yellow, PDB 2m6z) and docked structure of mTHPC in the heme-binding site of Hb. (**B**) Energy components of ΔE_binding_. (**C**) ΔE_binding_ decomposed per residue. (**D**) Graphical representation of the interaction between His58, Val62, Leu86, His87, Leu91, Val93, and mTHPC. Images created with VMD [73].

mTHPC occupies the heme-binding pocket, superimposing perfectly with the crystallographic structure of the heme (Figure 2A). The favourite binding site is located in the α-subunit, consistent with the fact that α chains in apohemoglobin dimers αβ have a higher affinity for heme than β chains [72]. The calculated ΔE_binding_ of mTHPC to Hb is −55.4 kcal mol^−1^. The driving force of the binding are van der Waals interactions (Figure 2B). Heme-binding pockets are hydrophobic environments, rich in aromatic and aliphatic amino acids. The presence of a hydrophobic pocket surrounding the heme is necessary for two reasons: (i) to provide a favorable environment to bind the hydrophobic heme, and (ii) to bind oxygen reversibly without going through oxidation or other undesired processes. The aromatic ring of mTHPC is buried among the hydrophobic amino acids of the protein (Val62, Leu86, Leu91, Val93) that usually bind the hydrophobic portions of the heme (Figure 2C,D). In Hb, anchoring of the heme is facilitated by a histidine nitrogen, His 58 (distal), that coordinates the iron. A second histidine is near the bound oxygen, His 87 (proximal). In mTHPC, the iron atom and the oxygen molecule are absent; the distal and proximal histidines are not involved in coordination and can interact with mTHPC via hydrogen bonds (electrostatic interactions in the MM-GBSA model).

**Figure 3:**
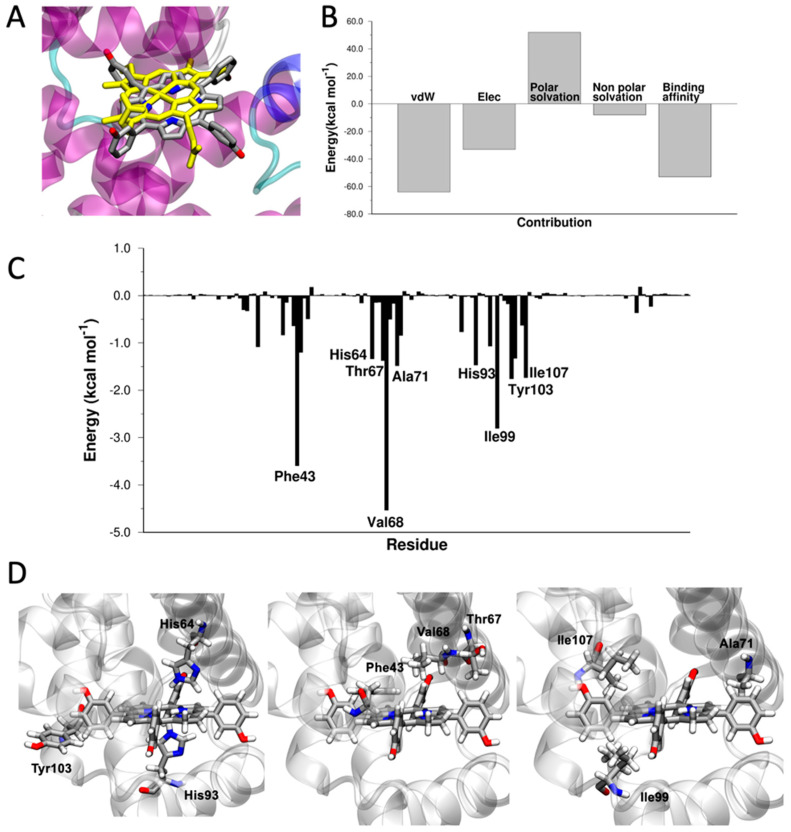
(**A**) Superposition of the crystallographic structure of heme (in yellow, PDB 3rgk) and docked structure of mTHPC in the heme-binding site of Mb. (**B**) Energy components of ΔE_binding_. (**C**) ΔE_binding_ decomposed per residue. (**D**) Graphical representation of the interaction between Phe43, Val68, Ala71, His93, Ile99, Tyr104, Ile107, and mTHPC. Images created with VMD [73].

Myoglobin (Mb) is an O_2_-binding hemoprotein, responsible for the storage of oxygen in muscles [52]. The stronger affinity for oxygen of myoglobin, with respect to hemoglobin, determines the diffusion of oxygen from the blood capillaries to the muscular tissues [52]. Mb is a single-chain monomeric protein composed of 153 amino acids (~17 kDa). Additionally, myoglobin shows the typical globin fold, consisting of eight α-helices [52]. Despite the difference in the kind and number of amino acids present, Mb shares almost identical secondary and tertiary structures with the α and β subunits of Hb. Similarities also includes the location and the chemical characteristics of the heme-binding pocket. In fact, both the total binding energy of mTHPC to Mb (ΔE_binding_ = −53.3 kcal mol^−1^) and the energy components of ΔE_binding_ strongly resemble those of Hb (Figure 3). The aromatic ring of mTHPC is accommodated in the same way of the heme (Figure 3), sandwiched by π-π interactions with Phe43 and Tyr103 and hydrophobic interactions with the aliphatic side chains of Val68, Ala71, Ile99, and Ile107 residues. The two distal (His64) and proximal (His93) histidine residues interact via hydrogen bonding with mTHPC (Figure 3).

The rigid structure of apoHb and apoMb, characterized by a specific, narrow, and well-defined heme-binding site, represents a great potential in bio-applications, especially as a natural protein carrier.

The third position of the rank is occupied by hemopexin (Hx). Human plasma hemopexin is a monomeric protein consisting of 439 amino acids (~60 kDa) [74]. The heme-binding site is located between two similar four-bladed β-propeller domains connected by an interdomain linker peptide [74]. Hemopexin is the protein with the highest affinity to heme among known proteins (K_d_ < 1 pM). Hx binds to the free hemes present in the blood, protecting against their cell-damaging effects and promoting their detoxification. After binding, hemopexin transports heme to the liver, which, after the interaction with specific receptors, releases it for breakdown and iron recovery [74]. The composition and location of the heme-binding pocket confers hemopexin high-affinity, yet reversible, binding to the heme. The high affinity for heme is obtained by the simultaneous iron coordination by two histidines (the binding site of Hx is rich in histidines), hydrophobic packing, and steric complementarity around the heme [74]. A notable characteristic of the heme-binding pocket in Hx is the preponderance of aromatic residues: of the eight hydrophobic residues forming the pocket, seven are aromatic [74]. Relative movements of the two domains and/or of the linker peptide connecting them break up the heme-binding pocket, causing its release [74]. It is the tridimensional organization of the two β-propeller domains, connected by an interdomain linker peptide, that defines a modular binding pocket, which contributes both to the high-affinity binding and/or release of the heme [74].

**Figure 4:**
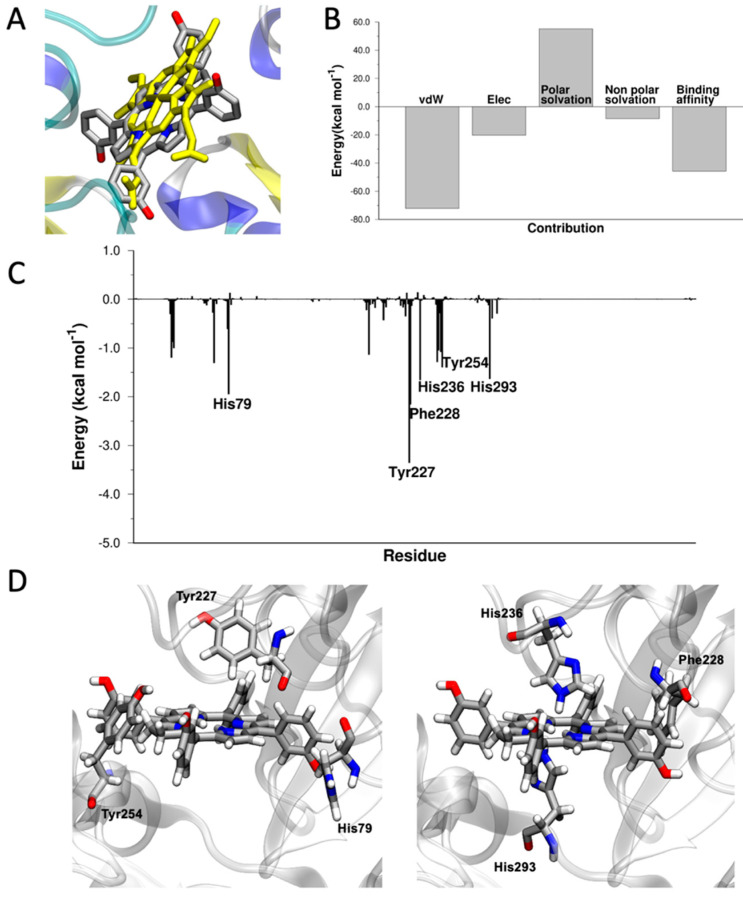
(**A**) Superposition of the crystallographic structure of heme (in yellow, PDB 1qjs) and docked structure of mTHPC in the heme-binding site of hemopexin. (**B**) Energy components of ΔE_binding_. (**C**) ΔE_binding_ decomposed per residue. (**D**) Graphical representation of the interaction between His79, Tyr227, Phe228, His236, Tyr254, His293, and mTHPC. Images created with VMD [73].

mTHPC binds in the heme-binding site of Hx (Figure S1), with a ΔE_binding_ of 45.7 kcal mol^−1^, strongly interacting with aromatic residues, such as Tyr227, Phe228, and Tyr254, and histidines (His79, His236, His293) (Figure 4).

The binding of porphyrins and PS to hemopexin has already been observed, suggesting the feasibility to use this protein as an mTHPC carrier [75,76,77,78].

The fourth position of the rank is occupied by afamin. Afamin resembles serum albumin in both structure (Figure S2) and functionality [52]. It is a carrier for hydrophobic molecules in body fluids, in particular for vitamin E [79]. It is a single-chain protein consisting of 578 amino acids. It has a molecular weight of 87 kDa, but in contrast to albumin, it is highly glycosylated, and treatment with N-glycanase reduces its MW to 65 kDa. The predicted binding site of mTHPC in afamin is the same identified for HSA, i.e., the cleft region (Figure S2).

Additionally, the ΔE_binding_ between mTHPC and either one of the two proteins is similar (ΔE_binding_ = 31.0 kcal mol^−1^ for afamin and ΔE_binding_ = 36.4 kcal mol^−1^ for HSA).

**Figure 5:**
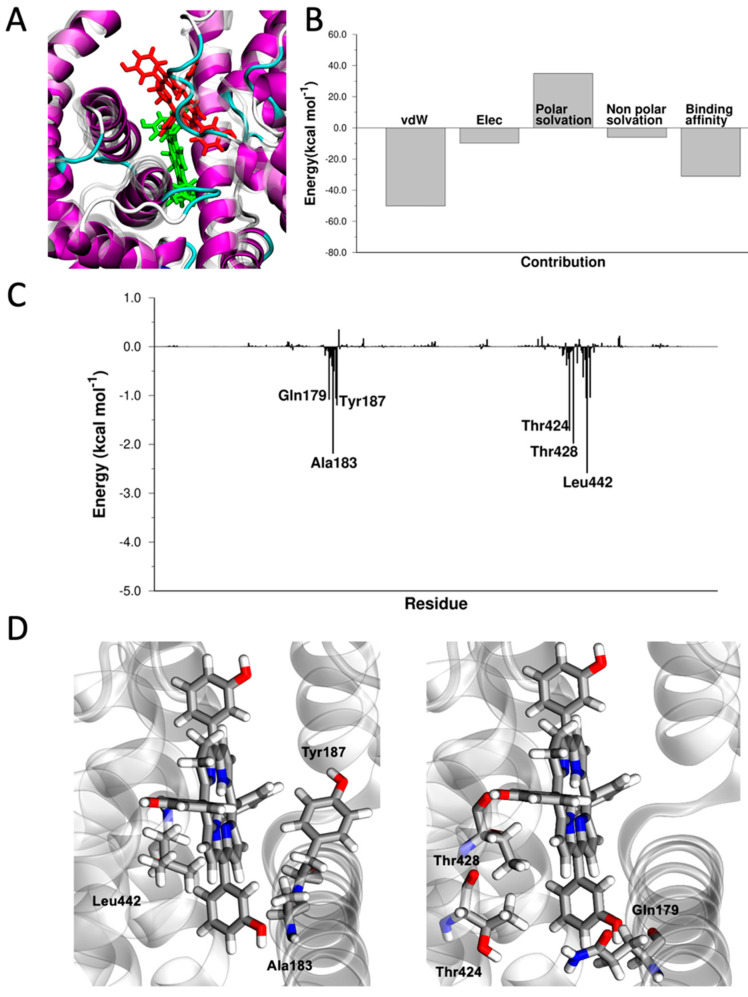
(**A**) Superposition of the docked structure of mTHPC in afamin (in green) and in HSA (in red). (**B**) Energy components of ΔE_binding_. (**C**) ΔE_binding_ decomposed per residue. (**D**) Graphical representation of the interaction between Gln179, Ala183, Tyr187, Thr424, Thr428, Leu442, and mTHPC. Images created with VMD [73].

Van der Waals interaction between the residues present in the cleft and mTHPC occur; in particular, mTHPC strongly interacts with Gln179, Ala183, Tyr187, Thr424, Thr428, and Leu442 (Figure 5). The dispersion of mTHPC by human serum albumin (HSA) has already been discussed from a computational and experimental point of view [68]. Of course, the calculated binding energy of mTHPC with afamin and HSA is considerably lower than Hb, Mb, and Hx because these proteins possess a specific pocket for heme-binding.

### 3.2. Synthesis and Characterization of the mTHPC@apoMb Complex

**Figure 6:**
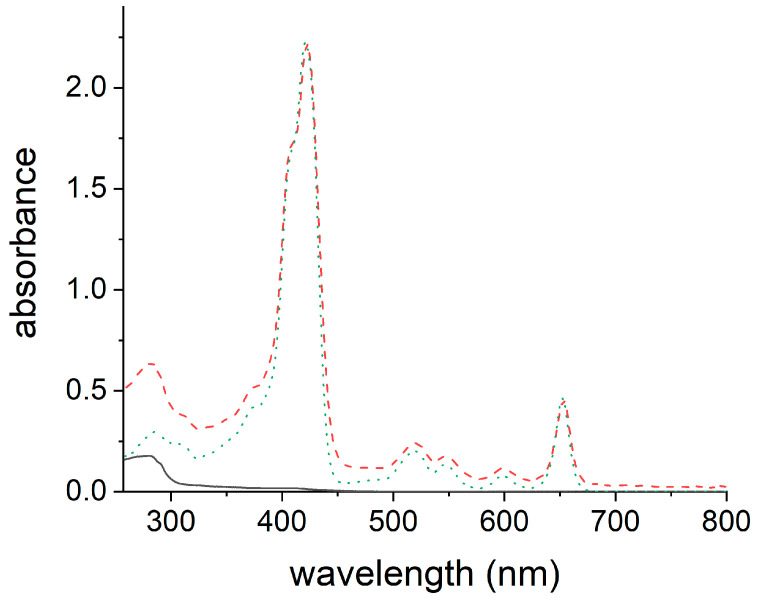
UV-Vis spectra of mTHPC@apoMb (dashed red line) in PBS, apoMb (solid black line) in PBS, and mTHPC in DMSO (dotted green line).

To demonstrate the possibility of using the blood transport proteins as mTHPC carriers, we selected myoglobin for experimental studies. The calculated affinity of mTHPC to Hb and Mb, as well as the structure of the heme-binding proteins, is practically the same, but it is simpler to handle myoglobin due to its monomeric nature. The prosthetic heme groups of Hb and Mb may be synthetically removed [61,80], producing, respectively, apohemoglobin (apoHb) and apomyoglobin (apoMb), heme-binding apoproteins with high affinity to the heme. ApoHb and apoMb have already been used in biomedical applications as delivery systems [81], in catalysis [82,83], and in nanobiotechnology [84]. Here, we prepared apomyoglobin (apoMb) using a standard procedure of extraction of the heme group with 2-butanone in acid conditions [61]. The heme is non-covalently bound in myoglobin (b-type heme); after acidification, its interaction with the protein is strongly reduced due to the protonation of its carboxylic groups. The transfer of the neutral heme from the aqueous solution to the organic phase was observed upon the addition of 2-butanone. To reconstitute myoglobin, a solution of mTHPC in 2-butanone was added to the apoMb. Alkalization of the solution induces deprotonation of the phenolic group of mTHPC and migration of the negatively charged mTHPC from the organic phase to the aqueous solution, leading to the reconstitution of myoglobin with mTHPC as a guest (mTHPC@apoMb). The mTHPC@apoMb complex was then purified by dialysis. The UV-Vis spectrum of mTHPC@apoMb (Figure 6) showed the success in the reconstitution of the Mb. In fact, it revealed the typical absorption band of the protein (280 nm). It also evidenced the diagnostic absorption bands of mTHPC. They are the Soret band at 422 nm and the four Q bands in the range of 500–680 nm.

**Figure 7:**
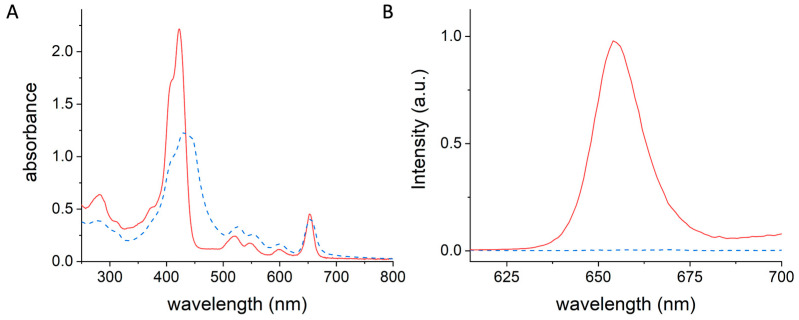
(**A**) UV-Vis absorption spectra and (**B**) emission spectra (λ_ex_ = 597 nm) of mTHPC@apoMb (solid red line) and mTHPC in PBS (dashed blue line).

mTHPC@apoMb in PBS displays the typical UV-Vis spectrum of mTHPC monomers (Figure 7A, solid red line), keeping the well-defined shape of the Soret band (422 nm) and the four Q-bands (500–680 nm) [85].

On the other hand, the broad and red-shifted Soret band, centered at 430 nm, which appears in the spectrum of the dispersion of mTHPC in PBS from a stock solution in DMSO (Figure 7A, dashed blue line), revealed that in this formulation, the majority of mTHPC exists in aggregated form.

The photosensitizing and imaging capabilities of mTHPC are significantly influenced by the aggregation state of mTHPC. The aggregated state dramatically reduces the fluorescence of mTHPC and its ability to produce ROS, due to quenching phenomena that occur in the aggregates [86,87,88]. Conversely, mTHPC monomers exhibit a significantly higher fluorescence emission and are unaffected by quenching [86,87,88].

The typical emission of mTHPC at 655 nm is observed for the mTHPC@Mb complex (Figure 7B), where mTHPC is monomolecularly dispersed, whereas an isoabsorbing solution of mTHPC in PBS does not show any emission signal due to aggregation (Figure 7B). The electrophoretic analysis of mTHPC@apoMb on agarose gel in native conditions clearly shows that the protein contains encasulated mTHPC. Whereas mTHPC alone does not show any emission signal (line A, Figure S3) due to aggregation, in the mTHPC@apoMb complex, the fluorescent spot of mTHPC superimposes with the spot of the protein (line B, Figure S3) following Coomassie staining, demonstrating the simultaneous presence of mTHPC and protein.

### 3.3. Evaluation of the PDT Performances of the mTHPC@apoMb Complex

**Figure 8:**
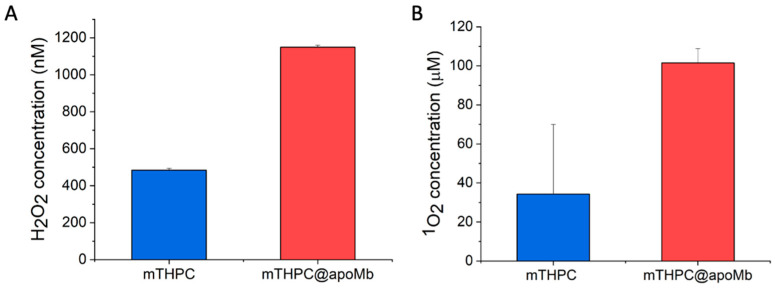
Quantification of ROS generated through (**A**) type I mechanism using Amplex Red assay for H_2_O_2_ detection and (**B**) type II mechanism using ABMDMA assay for ^1^O_2_ detection.

The effect of the encapsulation inside myoglobin on the photosensitizing properties of mTHPC was evaluated using 9,10-Anthracenediyl-bis(methylene)dimalonic acid, ABMDMA, and Amplex Red assays (Figure 8). These tests determine, respectively, the generation of singlet oxygen (type II mechanism) and peroxides (type I mechanism) upon irradiation.

The performances of the mTHPC@apoMb are significantly higher than mTHPC. The encapsulation of mTHPC improves both type I and type II ROS generation mechanisms.

In the case of type II mechanism, (i) the monomolecular dispersion of mTHPC inside the protein eliminates the well-known phenomenon of aggregation-caused quenching (ACQ), typical of many photosensitizers [22]; and (ii) the rapid quenching of the singlet oxygen produced by water molecules is reduced by the hydrophobic environment [89] of the heme-binding pocket, improving the production of ^1^O_2_ by ~300%.

In the case of the type I mechanism, an additional factor boosts the generation of ROS, which is the presence of electron-rich protein residues in Mb that take part in the electron transfer process necessary to generate oxygen radicals. The type I mechanism is generally activated by sacrificial electron donors; in the case of the mTHPC@apoMb complex, the process is self-activated by the protein itself [22,33,62,63,64,66,67,68].

**Figure 9:**
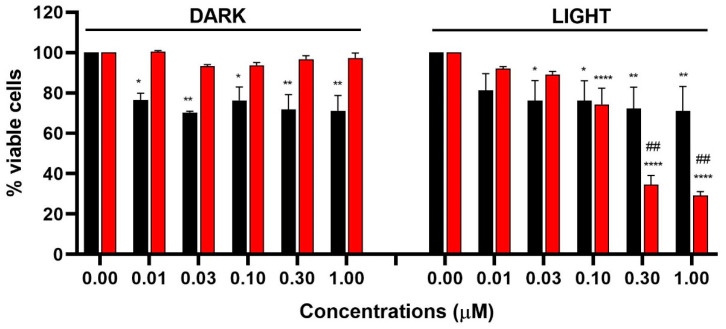
Cell viability of CAL27 cells after 24 h from 45 min treatment with increasing concentrations of mTHPC/CM (black bars) or mTHPC@apoMb (red bars), and in the presence or absence of 45 min of light irradiation. Statistical significance was calculated by one-way ANOVA followed by Dunnett’s multiple comparisons test or *t*-test. * *p* < 0.05, ** *p* < 0.001; **** *p* < 0.0001 compared to untreated cells; ## *p* < 0.01 compared to the same concentration of treatment with mTHPC/CM after light exposure.

We compared the cytotoxic and phototoxic potential of mTHPC@apoMb complex with mTHPC/CM, i.e., mTHPC dissolved in DMSO and diluted in complete medium (mTHPC/CM) (Figure 9). CAL27 cells were selected because they are one of the most frequently used cell lines in the field of head and neck cancer, for which mTHPC is approved.

In dark conditions, we observed the absence of cytotoxic effects for the monodispersed formulation of mTHPC@apoMb, whereas we recorded a significant decrease in cell viability after treatment with the mTHPC/CM at all tested concentrations (Figure 9). These cytotoxic effects may be due to the formation of aggregates that impaired cell viability. Upon irradiation, the phototoxicity of mTHPC/CM is negligible, whereas the phototoxic effects of mTHPC@myo significantly increase in a concentration-dependent manner (Figure 9). The calculated IC_50_ (inhibitory concentration causing 50% of cell death) of the irradiated mTHPC@apoMb is 0.24 µM.

In conclusion, we used virtual screening to identify blood transport proteins able to bind mTHPC. Apohemeproteins, such as apohemoglobin and apomyoglobin; the heme “scavenger” protein hemopexin; and two of the proteins responsible for the transport of hydrophobic molecules in the blood, i.e., afamin and human serum albumin, were identified. We developed a synthetic procedure able to replace the heme group with mTHPC in myoglobin. The protein confers to mTHPC (i) water solubility, (ii) monodispersity, (iii) and stability in physiological environments. We then validated the computational results using apomyoglobin as a true “Trojan horse” inside cancer cells. The mTHPC@apoMb complex represents a novel protein-based phototheranostic platform that preserves the imaging properties of mTHPC and, at the same time, improves its ability to generate ROS via both type I and type II mechanisms. In vitro experiments demonstrated that the new mTHPC@apoMb formulation is biocompatible and more effective than mTHPC in PDT.

## Figures and Tables

**Table 1:** Protein target candidates for mTHPC-binding, identified by reverse docking procedure. Only one protein structure, the one interacting the most with mTHPC, is indicated in the table.

Protein	PDB/UniProt ID	FireDock Score
Hemoglobin	2m6z	−70.1
Human myoglobin	3rgk	−67.4
Hemopexin	P02790	−65.2
Afamin	6fak	−59.9
Serum Albumin	6a7p	−53.2
Lactotransferrin	1lfg	−53.2
Plasma-retinol-binding protein	1qab	−51.5
Thyroxine-binding globulin	2ceo	−50.6
α-fetoprotein	J3KMX3	−50.3
Serotransferrin	6soy	−48.4
Vitamin-D-binding protein	1ma9	−47.7
Haptoglobin	4x0l	−44.4
Transthyretin	1g1o	−42.7
Ceruloplasmin	4ejx	−36.7
Sex-hormone-binding protein	6pyb	−34.3
Corticosteroid-binding globulin	2vdy	−27.1

## Data Availability

All data in this study can be requested from the corresponding authors (edoardojun.mattioli2@unibo.it, E.J.M.; and matteo.calvaresi3@unibo.it, M.C.)

## Supplementary Materials

The following supporting information can be downloaded at: https://www.mdpi.com/article/10.3390/pharmaceutics15030919/s1, Figure S1: Superposition of the crystallographic structure of heme and docked structure of mTHPC in Hx; Figure S2: mTHPC-binding pocket in afamin and in HSA; Figure S3: The agarose gel electrophoresis of mTHPC and mTHPC@apoMb.
