# Supplementary material for: Identification of Blood Transport Proteins to Carry Temoporfin: A Domino Approach from Virtual Screening to Synthesis and In Vitro PDT Testing
Source: Pharmaceutics. 2023 Mar 11;15(3):919. doi: 10.3390/pharmaceutics15030919 (PMC10056000; doi:10.3390/pharmaceutics15030919)
Supplement: Supplementary file 1 [file pharmaceutics-15-00919-s001.zip › pharmaceutics-2244496-supplementary.pdf]

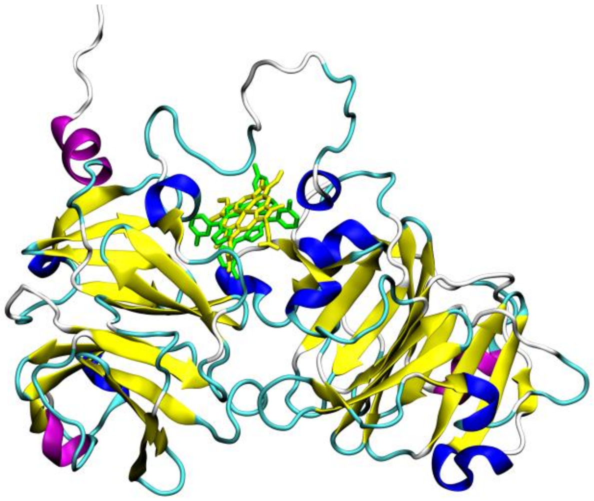

Figure S1. Superposition of the crystallographic structure of heme (in yellow, PDB 1qjs) and docked structure of mTHPC (in green) in the heme binding sites of Hx. Image created with VMD.

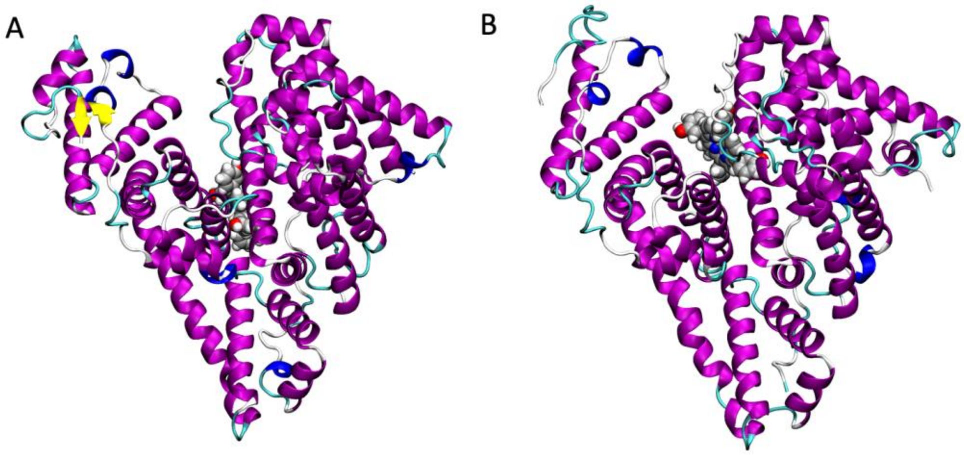

Figure S2. mTHPC binding pocket in (A) afamin and in (B) HSA, identified by the docking protocol. Images created with VMD.

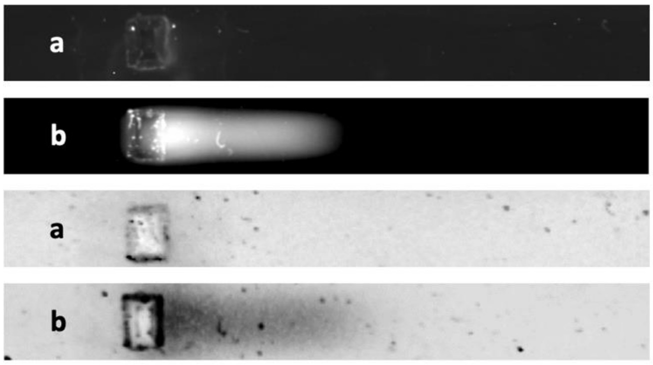

Figure S3. The agarose gel electrophoresis of (a) mTHPC, and (b) mTHPC@apoMb. The gel images were acquired before staining, in fluorescent mode (top), and after staining with Coomassie blue, in colorimetric mode (bottom).
